# Spontaneous Development of Immune-Mediated Necrotizing Myopathy (IMNM) After Chronic Use of Statins: A Case Report

**DOI:** 10.7759/cureus.42266

**Published:** 2023-07-21

**Authors:** Michael T Luong, Paul Danahy, Gerald Y Ho

**Affiliations:** 1 Orthopaedic Surgery, Lake Erie College of Osteopathic Medicine, Bradenton, USA; 2 Rheumatology, Arthritis and Osteoporosis Medical Center, Monterey Park, USA

**Keywords:** idiopathic inflammatory myopathies, anti hmg coa reductase, weakness, muscle necrosis, ivig, statin, imnm, immune mediated necrotizing myopathy

## Abstract

Statin medications, in addition to lifestyle modifications, have been the regimen of choice for addressing dyslipidemia in the general population. Its widespread use has been justified by increasing evidence that hyperlipidemia is a strong risk factor for the development of atherosclerotic disease resulting in myocardial infarction and other cardiovascular events. Unfortunately, this medication is not tolerated by some patients as it causes uncomfortable side effects such as myalgias, arthralgias, and headaches to name a few.

On rare occasions, some patients may develop immunity against the medication itself resulting in a condition known as immune-mediated necrotizing myopathy (IMNM). In such instances, patients carry antibodies against 3-hydroxy-3-methylglutaryl-coenzyme A reductase (HMGCR) or signal recognition particle (SRP). A small subset of patients may develop IMNM even in the absence of these two antibodies and they are termed seronegative. In this care report, we review the case of a 55-year-old Hispanic male with a history of Hashimoto's thyroiditis and hyperlipidemia who presented to an outpatient rheumatology office for severe proximal muscle weakness after being asymptomatic on rosuvastatin for over 20 years. The patient was stabilized with high-dose steroids and was subsequently given a regimen of mycophenolate and intravenous immunoglobulin (IVIG). He was able to regain approximately 75%-80% of his baseline muscle strength.

## Introduction

Immune-mediated necrotizing myopathy (IMNM) falls under the umbrella of idiopathic inflammatory myopathies (IIMs). More recognizable conditions under this umbrella include dermatomyositis and polymyositis among others. IMNM is divided into three subclasses based on the autoantibody found in the patient’s blood. These antibodies include 3-hydroxy-3-methylglutaryl-coenzyme A reductase (Anti-HMGCR), signal recognition particle (SRP), or autoantibody-negative [1]. Of the three subclasses, anti-HMGCR-induced IMNM is associated with the use of statin medications, but it is important to note that patients can develop these anti-HMGCR autoantibodies even without a history of statin usage [2]. Anti-HMGCR-induced IMNM is characterized by progressive muscle weakness, muscle biopsy demonstrating a lack of inflammatory infiltrations, and possible elevated creatine phosphokinase (CPK) [3]. Extra-muscular manifestations of Raynaud's phenomenon and interstitial lung disease in anti-HMGCR-induced IMNM are less commonly seen in comparison to the anti-SRP subclass [3]. Due to the rarity of the condition, no optimum or definitive treatment regimen has been established. As per previous clinical case reports, immunosuppressants and/or intravenous immunoglobulins (IVIG) have shown great promise [3]. We present a case of anti-HMGCR-induced IMNM that spontaneously developed in a patient with a history of statin use; a positive outcome was achieved utilizing a combination of mycophenolate, IVIG, and prednisone taper.

## Case presentation

A 55-year-old Hispanic male was referred to the clinic by his primary care provider. He had been discharged from the hospital after visiting the emergency room due to severe muscle weakness and an elevated CPK. The patient was stabilized on prednisone 60 mg once daily while inpatient and was discharged home to follow up with rheumatology. During the office visit, the patient showed mild improvement, however, his weakness was still present. He described his weakness as generalized fatigue with decreased muscle strength, making it difficult for him to lift his legs while walking on an even walkway or ascending stairs. The onset of his symptoms began over the last year but progressed rapidly over the last four months. Aside from his weakness, the patient denied any pain in the finger joints, wrist joints, hips, or knees. He furthermore denied any rashes or numbness. His past medical history was significant for hyperlipidemia, Hashimoto thyroiditis, and osteoarthritis. His home medications included rosuvastatin 10 mg, levothyroxine sodium 150 mcg, and vitamin D (cholecalciferol) 50 mcg. Physical examination was significant for an elevated blood pressure of 165/89 mmHg without a history of hypertension. Manual muscle testing (MMT) revealed 4-/5 on the left thigh flexion and 4+/5 on the right thigh flexion. Bilateral upper extremity muscle strength was 5/5. His gait appeared to be abnormal as there was notable mild circumduction of the left leg during the swing phase. 

Initial management included reducing prednisone to 40 mg once daily and initiating mycophenolate sodium 500 mg twice daily. Rosuvastatin was discontinued to prevent further exacerbation of the patient’s myositis. A muscle biopsy of the left thigh, magnetic resonance imaging (MRI) of the left thigh, myositis panel, and routine labs were ordered. In the process of obtaining records of the patient’s inpatient/ED course, it was discovered that the patient had the MRI and muscle biopsies ordered/completed inpatient. The results of the MRI were read by the hospital radiologist; the muscle biopsy was not yet available at the time of the initial office visit, but it became available one week later after. As both the MRI and muscle biopsy were completed recently, we used these results instead of completing new procedures. The initial lab results are mentioned in Table 1. 

**Table 1:** Results of Initial Laboratory Testing

Test	Result	Reference Range
Creatinine	0.7	0.5 - 1.1 mg/dL
Glomerular filtration rate (GFR)	115	>90 mL/min
Aspartate aminotransferase (AST)	226	<37 U/L
Alanine Transaminase (ALT)	399	<40 U/L
Creatine phosphokinase (CPK), Total	7043	24 – 170 U/L
Creatine Kinase MB	>300	0 – 5.00 ng/mL
Erythrocyte Sedimentation Rate (ESR)	7	<20 mm/hr
C-Reactive Protein (CRP)	<6	<6 mg/L
Antinuclear Antibodies (ANA)	Negative	Negative
Sjogren's Syndrome A (SS-A) Antibodies	0.12	<1.0
Sjogren's Syndrome B (SS-B) Antibodies	0.16	<1.0
Double-stranded DNA Antibody (Anti-DsDNA)	0.08	<0.90
C-3 Complement	128	90 – 180 mg/dL
C-4 Complement	33	10 – 40 mg/dL
Anti-Smith/Antiribonucleoprotein (Anti SM/RNP)	0.17	<1.0
Antihistidyl Transfer Synthase (Anti-JO-1)	Negative	<1.0
Chromatin Antibody	<0.2	0.0 - 0.9
Nuclesome remodeling-deacetylase (NuRD) complex (Mi-2)	Not detected	Not detected
Anti-signal recognition particle (SRP) antibodies	Not detected	Not detected
Aldolase, Serum	69.7	<8.1 U/L
3-hydroxy-3- methylglutaryl-coenzyme A reductase (HMGCR) Autoantibodies	151	Not detected

The results of the MRI of the left thigh and left thigh biopsy are as follows: 

MRI of the left thigh: There was muscle edema of the left rectus femoris, adductor brevis, abductor magnus, biceps femoris, semimembranosus, and semi-tendinosis muscle, and to a lesser extent, muscle edema of the vastus lateralis, vastus medialis, vastus intermedius, gluteus maximus, obturator internus, obturator externus, pectineus, and quadratus femoris muscles. Findings may be related to polymyositis, posttraumatic injury/strain/interstitial tears, and delayed onset muscle soreness. 

Left thigh muscle biopsy: The muscle biopsy sample showed extensive myofiber necrosis with regeneration and influx of myo-invasive macrophages with clearing of necrotic debris. No vasculitis was detected.

The left femur demonstrated a focal intramedullary lesion measuring 2.0 x 1.7 x 3.4 cm. The lesion itself is a narrow zone of transition with no associated cortical breakthrough or periosteal reaction and therefore, may represent a nonaggressive lesion. However, this cannot be determined with certainty and further evaluation such as with radiography/CT, PET scan, nuclear scintigraphy bone scan, and possibly bone biopsy will have to be considered. 

Follow up

After the muscle biopsy, left thigh MRI and HMGCR antibody results came back positive, the patient was diagnosed with anti-HMGCR-induced immune-mediated necrotizing myopathy. Additional labs also indicated an elevated high sensitivity thyroid-stimulating hormone (TSH) value of 89.10 uIU/mL (ref 0.27 - 4.20) but these findings were non-contributory due to the new and rapid progression of the muscle weakness and the concurrent thyroid replacement regimen that the patient was currently prescribed by their primary care provider. Between November 11, 2021 to May 05, 2022, the patient had a follow-up visit every 2-3 weeks. At each office visit, MMT was performed to monitor for improvement/deterioration of the patient’s muscle strength (Table 2).

**Table 2:** Manual Muscle Testing (MMT) Results X = Value was not recorded or test was not performed.

	11/18/21	12/09/21	12/22/21	01/21/22	03/03/22	04/07/22
Right Upper Extremity	5	4	4	5	5	5
Left Upper Extremity	5	4	4	5	5	5
Right Hip Flexion	4+	3+	3+	4-	4-	4
Left Hip Flexion	4-	3+	3+	4-	4-	4
Right Hip Extension	x	x	x	3+	3+	x
Left Hip Extension	x	x	x	3+	3+	x

In addition to MMT, CPK levels were also drawn during each office visit to monitor for worsening of myositis and improvement in treatment regimen that may not be apparent on MMT. The trend of the CPK lab values is shown in Table 3. 

**Table 3:** Creatine Phosphokinase (CPK) Trend

Date	11/22/21	12/06/21	12/20/21	01/17/22	02/24/22	04/04/22	05/05/22
CPK, Total	7043 U/L	6458 U/L	4421 U/L	3164 U/L	180 U/L	110 U/L	118 U/L

The first three follow-up visits showed a decline in muscle strength as his right hip flexion decreased from 4+ to 3+. Strength of the left hip flexors decreased in the same period from 4- to 3+. CPK values from the first three visits showed improvement from 7043 U/L initially to 4421 U/L. The patient's mycophenolate increased during this time from 500 mg twice daily to 1000 mg three times daily. Prednisone was increased back to 60 mg once daily. With the patient's muscle strength worsening, he was started on IVIG 2g/kg per month (beginning in January 2022) which resulted in a marked improvement in his condition. The final four follow-up visits showed that his hip flexors improved from 3+ to 4 bilaterally on the MMT. CPK values normalized within the first month after starting IVIG and have remained in the normal range from February 24, 2022 to May 5, 2022. The patient's prednisone dosage has been slowly tapered down to 5 mg once daily with plans to discontinue it in the near future; mycophenolate has been reduced to 1000 mg twice daily. The patient’s blood pressure readings have been in the normal range during all of the follow-up office visits.

Outcome 

The patient’s muscle weakness improved in comparison to his initial presentation to the clinic. Although the combination therapy of prednisone, mycophenolate, and IVIG halted the progression of the patient’s muscle weakness, it did not completely restore his muscle strength back to baseline. He was lost to follow up shortly after.

## Discussion

IMNM is a rare disease characterized by myofiber necrosis in the presence of minimal/absent inflammation histologically [3]. This condition can be classified into three categories based on the presence of antibodies against SRP, HMGCR, or autoantibody-negative [1]. Given their widespread use, a history of statin use could trigger the development of anti-HMGCR antibodies in the future, as seen in the case of this patient. However, it is possible for patients to also develop anti-HMGCR antibodies in the absence of statin-use history [2]. Anti-HMGCR-induced IMNM affects both genders equally and younger presentation of the disease is associated with a worse prognosis due to disease resistance to therapy [4]. Although there are no definitive treatment regimens for IMNM, the correct subclass of IMNM must be identified, as each subclass of IMNM has shown increased responsiveness to different treatment regimens. 

The treatment goals of IMNM are to improve the muscle strength of patients, halt the progression of the illness, normalize the creatine kinase values, and minimize the side effects of immunomodulators and glucocorticoids [4]. In all classes of IMNM, the first medication given is generally a glucocorticoid such as prednisone due to its anti-inflammatory properties. Glucocorticoids, when used alone, are generally insufficient to manage the disease [4]. In general, the next medication that is added is an immunomodulator but the selection of the immunomodulating agent is based on the autoantibodies present for the specific patient. Patients with anti-HMGCR respond very well to IVIG therapy with methotrexate which is given to augment treatment as needed. Patients with anti-SRP autoantibodies respond to the combination of Rituxan with methotrexate which is given to augment the treatment regimen [4-5]. For the patient in the case, his IMNM responded extremely well to IVIG. Rather than methotrexate, his therapy was augmented with the addition of mycophenolate which also provided an improvement in his disease course. 

Responsiveness to the therapy and long-term monitoring of the disease is done by utilizing a multi-modal approach. The patient’s motor strength is assessed in the office using MMT. This testing is used quite frequently in patients complaining of muscle weakness whether it is IMNM related or not. It can be used to evaluate the patient longitudinally. Some disadvantages to this method include the subjective interpretation of the examiner. In the case of our patient, this flaw was further increased as the examiner changed multiple times during the course of his care. Laboratory results also play an important role in the management of IMNM patients. Just as an increasing creatine kinase value can be interpreted as a worsening of the disease, an improving creatine kinase can be viewed as an improvement in the patient’s condition. In the case of IMNM, a normal creatine kinase value can be viewed as remission of IMNM [4]. Our patient achieved normal creatine kinase levels for three consecutive months qualifying him for remission status.

Of note, patients may not completely recover their baseline muscle strength. This may be due to their muscle fibers being replaced by fat during active disease [4]. This was also seen in our patient’s most recent muscle testing which showed thigh flexion/extension to be 4+ bilaterally despite CPK values being normal. This could be explained by following a normal skeletal muscle cell histologically as it is damaged by IMNM. Initially, there will be very minimal muscle necroses that are seen sporadically with mild changes in the cell nucleus. As IMNM progresses, the number of muscle cell necroses increases, cell nuclei become misshapen, and the endomysium layer between muscle cells is infiltrated by connective tissue and fat. All the above findings continue to become exaggerated as IMNM becomes more and more severe. Due to the connective tissue infiltration, this decreases the contractability of the muscle cells leading to weakness. Recalling that skeletal muscle cells are permanent cells that are post-mitotic, these changes are irreversible. Despite this, patients can continually improve their muscle strength with exercise and strength/resistance training which will hypertrophy the remaining muscle fibers. Clinicians should adjust the amount of weight and resistance to a level that is tolerated by the patient.

Limitations to this case report include an inability to obtain histological and MRI images. The decision to compose the case report was made retrospectively and thus the images were not requested when the results were received initially. Furthermore, the primary author of this paper resigned from his position on the patient’s healthcare team and is no longer active in the care of the patient. As such, communication with the hospital to obtain images has been handled by the patient’s rheumatology office. Unfortunately, images have yet to be received by the rheumatology office.

## Conclusions

IMNM is a rapidly progressing disease characterized by severe muscle weakness. Anti-HMGCR-induced IMNM is a subset of IMNM which is largely attributed to a history of statin use; however, patients do develop this illness without the use of statins. This case demonstrates a patient who developed the condition spontaneously after being asymptomatic on statin therapy for many years. Patients with autoantibodies against HMGCR can respond favorably to a therapy regimen consisting of prednisone, mycophenolate, and IVIG, as seen in our patient. A suggestion for further research would be to analyze the effects of insulin-like growth factor 1 or testosterone on the improvement of muscle weakness secondary to IMNM. Another research opportunity would be to investigate the best pharmacologic treatment modalities available to treat hyperlipidemia in patients with a past medical history of IMNM. 
